# Cashew apple in Tanzania: status of utilization, challenges, and opportunities for sustainable development

**DOI:** 10.12688/f1000research.124596.1

**Published:** 2022-11-21

**Authors:** Noel Dimoso, Neema Kassim, Edna Makule

**Affiliations:** 1 Department of Food Biotechnology and Nutritional Sciences, Nelson Mandela African Institution of Science and Technology (NM-AIST), Arusha, 23311, Tanzania

**Keywords:** cashew apples, utilization status, sustainable development, Tanzania

## Abstract

Cashew apples, although widely available and rich in nutrients are still underutilized after harvest in Tanzania. Approximately 2,327,000 metric tonnes of cashew apples are lost each year. Their counterpart, the cashew nut is highly appreciated and successfully contributes to the national economy. The huge underutilization of cashew apples is a challenge that requires urgent attention in order to achieve both national and global Sustainable Development Goals (SDGs) by 2030. Improvement of the cashew apple sector could have a positive impact on global SDGs 1, 2, and 3 targets of no poverty, zero hunger, and good health and well-being respectively. At national level, this sector could contribute to the goals of the Tanzania Development Vision (TDV) 2025, namely: high quality livelihood, particularly food self-sufficiency and food security; and a strong and competitive economy, particularly a diversified and semi-industrialized economy with a substantial industrial sector comparable to typical middle-income countries. In addition, the country’s Third Five Year Development Plan III (FYDP III) 2021/22 – 2025/26 has established key strategic interventions, notably those related to competitive industrialization, energy, and food and nutrition security to ultimately achieve the goals of the TDV 2025. To be effective, however, these strategic interventions require continued strong central and local government support and active involvement of stakeholders to ensure program effectiveness yielded towards efficient utilization of widely available natural resources such as cashew apples which has cross-cutting benefits in food, agriculture, health, energy, and economic perspectives. Therefore, the work provides evidence on the utilization status, challenges, and opportunities for sustainable development in Tanzania.

## Introduction

**Figure 1.**
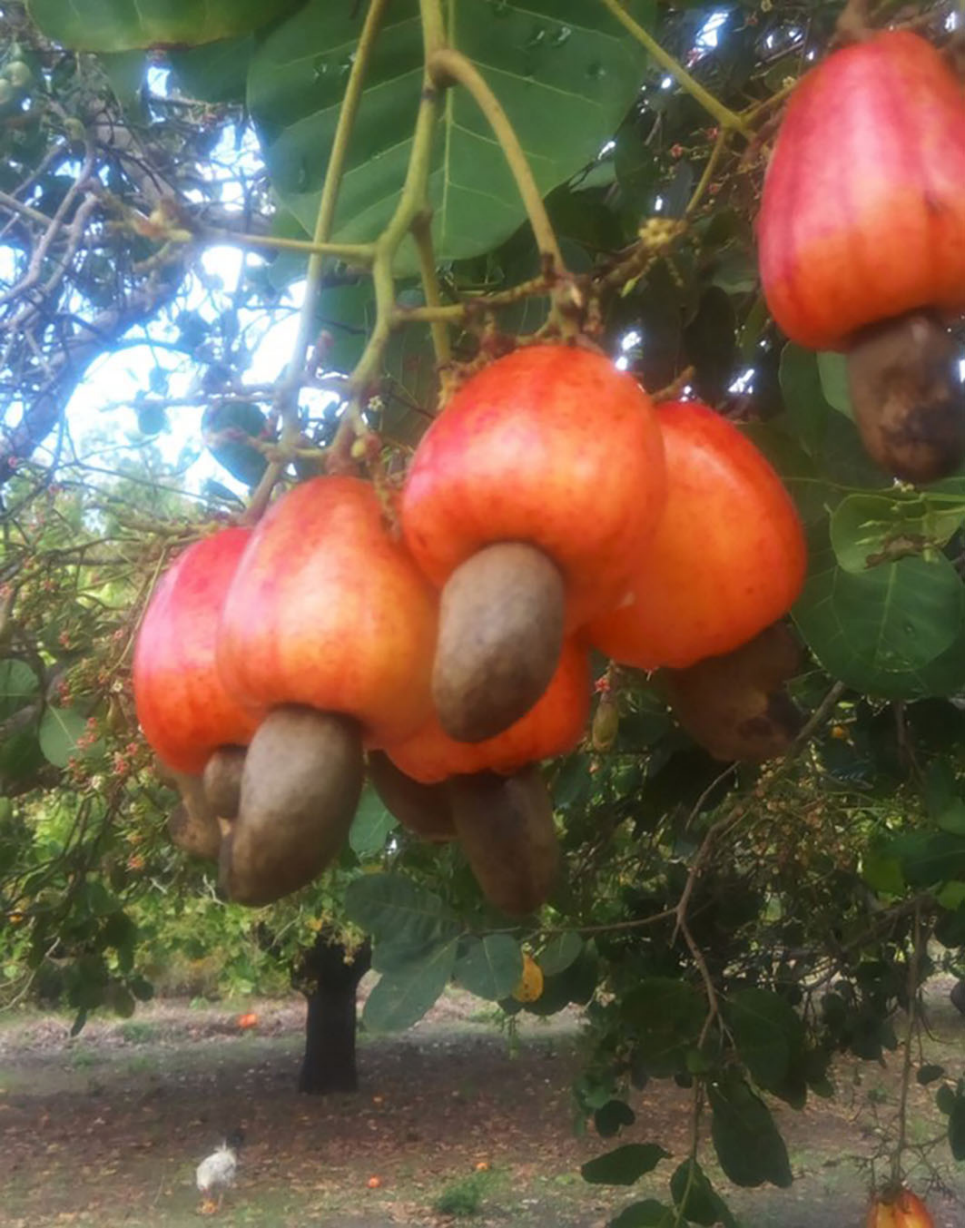
Hanging cashew nuts and cashew apples on a tree (Source: Authors).

The cashew sector is of great importance to the economy of Tanzania. As of 2015, the sector contributed around 497 billion Tanzanian Shillings, courtesy of cashew nuts ( Msoka *et al*., 2017). However, the cashew tree bears both the cashew nut and cashew apple ( Figure 1), and thus the latter is left to rot in the fields and less likely to be processed or fed to livestock. For instance, the estimated production of cashew apples in the country is about 2,327,000 metric tonnes, and yet there’s no significant commercial processing of this fruit ( Dimoso *et al*., 2021). Unexpectedly, utilization of this fruit has been for decades hampered by several factors: high perishability and astringent nature of the fruits; inadequate public knowledge and awareness of their potential for food and nutrition security and socio-economic development; inadequate skills and technologies for value addition; and weak investment and collaboration among stakeholders ( Dimoso *et al*., 2021). This fruit contains several important ingredients including vitamins (A and C), sugars, minerals, polyphenols and dietary fibers ( Msoka *et al*., 2017), hence it is suitable for processing of value-added products in food, bio-energy, pharmaceutical, and biochemical processing industries.

The huge underutilization of cashew apples is a challenge that requires urgent attention in order to achieve both national and global Sustainable Development Goals (SDGs) by 2030. Considering the country’s huge production of cashew apples as raw materials in different industrial sectors, adequate utilization of these fruits could have a positive impact on global SDGs 1, 2, and 3 targets of no poverty, zero hunger, and good health and well-being respectively ( United Nations, 2015). At national level, the cashew apple sector could contribute to the achievement of goals of the Tanzania Development Vision (TDV) 2025 namely: high quality livelihood, particularly food self-sufficiency and food security; and a strong and competitive economy, particularly a diversified and semi-industrialized economy with a substantial industrial sector comparable to typical middle-income countries. In addition, the country’s Third Five Year Development Plan (FYDP III) 2021/22 – 2025/26 has established key strategic interventions, notably those related to competitive industrialization, energy, and food and nutrition security to ultimately achieve goals of the TDV 2025 ( United Republic of Tanzania [URT], 2021). Similarly, the National Post-Harvest Management Strategy (NPHMS) 2019-2029 addressed strategic objectives aimed at reducing postharvest losses of agricultural crops with an ultimate goal of increasing stakeholders’ income and food and nutrition security ( URT, 2019). To be effective, however, these strategic interventions require continued strong central and local government support and active involvement of stakeholders to ensure program effectiveness yielded towards efficient utilization of widely available natural resources such as cashew apples which has cross-cutting benefits in food, agriculture, health, energy, and economic perspectives. Therefore, this policy brief provides local and international evidence regarding the quality of cashew apples (based on the study by Msoka *et al*., 2017), the utilization status and challenges (based on the study by Dimoso *et al*., 2021), product development interventions (based on the study by Dimoso *et al*., 2020) and addresses the need for strong policy and programmes in order to harness the full potential of cashew apples for sustainable development in Tanzania.

## Policy outcomes and implications

### Benefits of cashew apples

There are several feasible usages of the cashew apple ( Table 1), which if sufficiently exploited could help in part achieve the goals of the FYDP III and TDV 2025. For instance, in the arena of food and nutrition security, the FYDP III looks to reduce the prevalence of vitamin A deficiency among children aged 6-59 months to less than 20 percent, the proportion of women aged 15-49 years with anaemia from 44 to 22 percent, and reduction of acute malnutrition by year 2025, among others. The same micronutrient challenges were addressed by the Tanzania Demographic Health Survey 2010 ( URT, 2011). Increasing public awareness on the health benefits of cashew apple could increase their consumption (raw or processed form) and hence reduce a prevailing health burden. With regard to industrialization, the FYDP III emphasizes on value addition in agriculture through the use of science, technology and innovation as well as research and development. Since huge postharvest losses impact nutrition (especially on micronutrient deficiencies), the NPHMS outlines strategic interventions including facilitation of awareness on postharvest management, value addition, and improving agricultural marketing infrastructure to reduce losses particularly on perishable crops such as fruits and vegetables. For these reasons, cashew apple provides a diversified quality of products for domestic and export market, and hence stimulates socio-economic development.

**Table 1.** Multiple potential utilization of cashew apples.

Raw material	Potential sectors	Potential products	References
Cashew apples	Food	Fresh fruits	Paiva, 2012
		Fresh beverages (Juice, Syrup, Squash, Soda, Nectar)	Talasila *et al*., 2012
		Alcoholic beverages (Wine, Distilled liquor)	Gamero *et al*., 2019
		Probiotic beverages	Kaprasob *et al*., 2018
		Culinary products (Vinegar, Pickles, Preserve, Jam)	Silva *et al*., 2007
		Confectionary products (Candy, Biscuit)	Dimoso *et al*., 2020
		Cashew apple fibers/powder (added in soup, snack bars, cake)	Adegunwa *et al*., 2020
		Natural food additive (Color)	Paiva, 2012
	Energy	Bioethanol	Talasila and Vechalapu, 2015; Teles *et al*., 2017
		Microbial fuel cell (low voltage electricity)	Priya and Setty, 2019
	Biochemical processing	Lactic acid, Oxalic acid, Dextran, Mannitol, Oligosaccharides, Bio-surfactant	Nogueira *et al*., 2019; Betiku *et al.*, 2016
	Agriculture	Animal feed	Mathew *et al*., 2013
	Pharmaceutical	Nutraceuticals (Vitamin C, Polyphenols, Dietary fibers)	Ribeiro da Silva *et al*., 2014

Equally important, there is a severe unemployment burden in Tanzania, particularly among young people. A large segment (75 percent) of Tanzania’s population is under the age of 35 years. This young generation faces a great challenge to land employment immediately after finishing their qualifications. Statistically, out of one million young people, only 20 percent get employed immediately. In line with the FYDP III, an entrepreneurship approach is the feasible option to solve youths’ unemployment burden. Establishing and strengthening the Small and Medium Enterprises (SMEs) is critical due to the fact that, SMEs utilize locally available materials and have the potential to engage many people especially youth, women, and people with disabilities. There are many SMEs processing cashew nuts in cashew producing regions such as Mtwara, Lindi, Tanga, and Pwani, while not the same can be said about cashew apples. With the aforementioned benefits, cashew apple presents greater opportunities for researchers, politicians, investors and farmers to exploit its potentials, and ultimately drive forward national economy.

### Quality and utilization of raw cashew apples in Tanzania

Based on a recent study by Msoka *et al*. (2017), Tanzania’s cashew apple varieties are of considerable good quality. A study revealed a good quantity of valuable components such as vitamin C, carotenoid (provitamin A), sugars, polyphenols, dietary fibers, and minerals such as calcium, magnesium, sodium, potassium as well as low quantities of phosphorus, iron, and zinc. These ingredients make cashew apple a perfect raw material in food, energy, agriculture, pharmaceutical, and biochemical processing industries. From a health perspective, vitamin C, carotenoid, and polyphenols have antioxidant and anti-inflammatory activities, thus provide cashew apple an ability to reduce, prevent, or treat a number of chronic diseases such as scurvy, cancer, and neurodegenerative diseases. Since this fruit has a vitamin C content almost five times that of orange or mango, it can be used in food fortification and formulation. Furthermore, presence of sugars, dietary fibers, and minerals make cashew apple a perfect microbial substrate in different fermentation processes to produce value added products such as ethanol (as food drink or energy), organic acids, and bio-surfactant.

A field survey was conducted in major cashew producing regions i.e. Mtwara and Lindi to obtain insightful information regarding the utilization aspects of cashew apple ( Dimoso *et al*., 2021). The study revealed that the majority (98 percent) of cashew farmers consume raw cashew apples, with 62 percent consuming more than five fruits a day and about 56 percent consuming almost every day during the fruit season. However, farmers’ knowledge on the importance of eating cashew apple seemed limited. As a matter of fact, the majority of consumers (53 percent) claimed to eat cashew apple just because it is a fruit. In addition, few respondents seemed to acknowledge the contribution of fruits such as cashew apple to human health. Moreover, nearly 44 percent of farmers traditionally process cashew apple into porridge (traditionally called *Mkongohu*) and alcoholic drinks namely wine (traditionally called *Uraka*) and distilled liquor (traditionally called *Nipa*). With respect to challenges, lack of knowledge on postharvest handling (86 percent), and inadequate processing technologies (83 percent), among others were mostly claimed to hamper the utilization.

### Current modern processing and opportunities of cashew apple in Tanzania

Recently, Nelson Mandela African Institution of Science and Technology (NM-AIST) has formulated dried fruit slices and juice products from cashew apple ( Dimoso *et al*., 2020). The formulated cashew apple products were deemed acceptable with respect to nutrient retention, shelf-life stability and sensory properties. Similarly, Tanzania Agricultural Research Institute (TARI) - Naliendele and Ndanda Mission have also been developing cashew apple juice, jam and wine ( UNIDO, 2011). However, a commercialization stage of the aforementioned prototypes is yet to be attained.

It is apparent that, while few food products have been already developed, other value-added products for sectors such as bio-chemical, energy, and pharmaceutical industries remain unexplored. The current situation provides an opportunity for stakeholders to venture in cashew apple value chain. Therefore, the initiatives for product development and commercialization of cashew apple value-added products need to be formulated and implemented. This will foster the sustainable existence of cashew apple products in the market.

Value addition through agro-processing of highly perishable crops such as fruits and vegetables should be emphasized. According to the Ministry of Industry and Trade survey 2013, there were only 17 fruit processing units in a country, of which none is for cashew apples ( URT, 2019). Shortage of fruit processing industries account largely for the reported huge postharvest losses of such commodity. In this regard, the NPHMS has the following strategic objectives, among others that could benefit the cashew apple value chain: (1) promote availability, accessibility, affordability and adoption of tested technologies and processes to reduce post-harvest losses; (2) promote research and innovations of new and appropriate technologies and methods to reduce crop losses; (3) facilitate agricultural marketing systems to improve market access and minimize post-harvest losses. Likewise, value addition, food fortification and formulation, technology development and transfer, and commercialization of local agro-products are among the top research priorities outlined by the National Research Priorities 2021/22-2025/26 ( URT, 2022) to facilitate human capital development and building of strong and competitive industrial economy.

### International evidence on cashew apple processing

The most successful country in cashew apple processing is Brazil. This country has about 12 different cashew apple juice processing industries alone, while others sell fresh fruits, dried fruits, jam, wine, confectionaries, and animal feeds. Equally important, the Brazilian Agricultural Promotion Agency (EMBRAPA) is the leading actor in cashew apple value chain. For instance, it developed a cashew apple variety that can remain on the ground for 1 day without being damaged or beginning to ferment. On the contrary, Tanzanian varieties are very soft and get damaged once they fall on the ground, hence they require immediate processing and/or a cold chain facility for distant transportation or long-term storage. In addition, limited access to a sustainable cold chain for perishable crops (fruits and vegetables) is a dominant challenge not only in Tanzania but the whole region of Sub-Saharan Africa ( Makule *et al*., 2022). India has also progressed in developing cashew apple products, however, their commercialization is still a challenge.

However, in West African countries such as Ghana, Benin and Guinea-Bissau, there is little effort regarding cashew apple usage ( Monteiro *et al.*, 2017). Benin and Ghana have started producing and marketing cashew apple juice, although the sector is still in its infancy ( TechnoServe, 2017). According to Yantannou (2017), no country in Africa is processing greater than 1% of its cashew apple production. Therefore, there are greater business opportunities in the cashew apple value chain in African cashew producing countries.

## Actionable recommendations

Short-term recommended actions to be considered include: reinforcing access to affordable financial resources to all actors in cashew apple value chain; developing innovative and appropriate postharvest handling technologies to prolong cashew apple shelf life, and enhancing their adoption by relevant actors in the value chain; improving capacity building, extension services and access to information; designing and launching campaigns to raise awareness on the contribution of cashew apple in nutrition and income generation to farmers and all key players; and the realization of efficient utilization can be preceded by conducting market analysis, gaining understanding of consumer preference and strengthening the linkage between farmers and buyers.

Medium-term recommended actions include: strengthening research and development institutions to improve research outputs that aim to improve cashew apple breeds and value additions; creating a supportive environment for partnerships and collaborations among governmental and non-governmental actors to promote cashew apple projects; encouraging and supporting youth and women to venture into the cashew apple value chain; developing cost-effective cold storage technologies to prolong the shelf-life of perishable crops; and measuring the performance and benefits of all activities in the cashew apple value chain in order to generate knowledge, support learning, track progress, and ensure accountability.

## Conclusions

Addressing the wastage of cashew apples in Tanzania and the underlying opportunities is necessary in order to realize the goals of the FYDP III and TDV 2025. Among other factors, astringency and high perishability characteristics, inadequate processing skills and technology, and limited access to capital have contributed to the underutilization of these fruits. Being rich in vitamins, minerals, and bioactive components, cashew apples could be processed into a number value-added products. Increasing public awareness on the importance of cashew apples as a source of food and income could accelerate their utilization. Additionally, all stakeholders including individuals, agricultural research institutions and universities, and other public and private sectors are encouraged to participate in this endeavor to facilitate research, technology transfer and development, capacity building, and improve agricultural marketing infrastructure in order to utilize cashew apples effectively and efficiently and ultimately increase food and nutrition security and socio-economic development. Furthermore, all established national policies, strategies and programmes should be supported to implement the underlying objectives in relation to the cashew apple value chain.

## Data availability

### Underlying data

The raw data for the survey conducted by Dimoso *et al.* (2021) is restricted. Only members of the Fruits and Vegetables for all Season (FruVaSe) project can access the data directly. However, data can be accessed upon request by non-members. Please consult Dr Edna Makule via email ( edna.makule@nm-aist.ac.tz) to obtain the data file.

- Data on the physio-chemical quality of cashew apples in Tanzania: https://dspace.nm-aist.ac.tz/bitstream/handle/20.500.12479/183/JA_LiSBE_2017.pdf
- Data on the quality assessment of dried cashew apples: https://dspace.nm-aist.ac.tz/bitstream/handle/20.500.12479/1092/JA_LiSBE_2020.pdf
- Data on the utilization status of cashew apples in Tanzania was originally presented from our published research that can be accessed at: https://doi.org/10.1177/0030727020941164

These data are under open access and can be accessed at their respective links/DOI.

## Author contributions

**Dimoso N:** Conceptualization, Methodology, Investigation, Writing – Original Draft Preparation, Writing – Review & Editing; **Kassim N:** Supervision, Conceptualization, Writing – Review & Editing; **Makule E:** Supervision, Conceptualization, Funding Acquisition, Project Administration, Writing – Review & Editing.
