# Supplementary material for: Cashew apple in Tanzania: status of utilization, challenges, and opportunities for sustainable development
Source: F1000Res. 2024 Apr 2;11:1354. Originally published 2022 Nov 21. [Version 2] doi: 10.12688/f1000research.124596.2 (PMC11445638; doi:10.12688/f1000research.124596.2)
Supplement: Peer review history

# Reviewer report: Reviewer response for version 2

**DOI:** 10.5256/f1000research.164130.r325040

**Published:** 2024-10-01

**Authors:** Yusuf Momohjimoh

**Affiliations:** 1 Department of Agricultural Technology, Kogi State Polytechnic, Lokoja, Nigeria

The policy brief potentially reflects the utilization and challenges faced with cashew apple consumption, outlines the opportunity for sustainable development in Tanzania. The topic of discussion in this document is a significant one that contribute to the present-day research knowledge and aligned with current research trend. The paper being more of a narrative review employing case studies, did not report method of data collection from which inferences (results) are drawn. In most cases where data from research journal publications, case studies, newspapers, bureau of statistics, conferences, government bulletins amongst other are utilized, authors should specify appropriately. All sections have been appropriately stated. However typographical errors should be effected as corrected in the body of the work. Certain questions have been raised in the comment box in the body of the work (affected parts highlighted in yellow color), authors should look into them appropriately. Abstract section however requires major revision as it only talks about the under-utilization of cashew apple in Tanzania while highlighting the need for higher utilization to commensurate with the Tanzania Development Vision (TDV) 2025. Authors should revise the Abstract section to reflect the research topic while highlighting and/or summarizing the objectives of the paper, the methodology used, result obtained and conclusion/recommendation. Introduction section is well presented and substantiated with sufficient literatures. However, “Future perspectives on cashew apple processing” should be substantiated with sufficient literatures.

Does the paper provide a comprehensive overview of the policy and the context of its implementation in a way which is accessible to a general reader?

Yes

Is the discussion on the implications clearly and accurately presented and does it cite the current literature?

Yes

Are the recommendations made clear, balanced, and justified on the basis of the presented arguments?

Yes

Reviewer Expertise:

Agronomy, soil fertility, genetics and seed production, agricultural economics.

I confirm that I have read this submission and believe that I have an appropriate level of expertise to confirm that it is of an acceptable scientific standard, however I have significant reservations, as outlined above.

# Reviewer report: Reviewer response for version 2

**DOI:** 10.5256/f1000research.164130.r261901

**Published:** 2024-05-10

**Authors:** Sampson Kofi Kyei

**Affiliations:** 1 Associate Professor of Industrial Chemistry, Department of Chemical Engineering, Kumasi Technical University, Kumasi, Ashanti Region, Ghana

I Accept the manuscript in its current form.

 Thank you.

Does the paper provide a comprehensive overview of the policy and the context of its implementation in a way which is accessible to a general reader?

Yes

Is the discussion on the implications clearly and accurately presented and does it cite the current literature?

Partly

Are the recommendations made clear, balanced, and justified on the basis of the presented arguments?

Partly

Reviewer Expertise:

Waste and Biomass valorisation; Industrial Chemistry, Environmental Chemistry

I confirm that I have read this submission and believe that I have an appropriate level of expertise to confirm that it is of an acceptable scientific standard.

# Reviewer report: Reviewer response for version 1

**DOI:** 10.5256/f1000research.136803.r207201

**Published:** 2023-10-03

**Authors:** Sampson Kofi Kyei

**Affiliations:** 1 Associate Professor of Industrial Chemistry, Department of Chemical Engineering, Kumasi Technical University, Kumasi, Ashanti Region, Ghana

I found manuscript entitled "Cashew apple in Tanzania: status of utilization, challenges, and opportunities for sustainable development"

 very interesting. While going through the manuscript I found that manuscript is well-recognized to an extent and could be considered as a valuable contribution, but requires a minor revision. I would like to raise some comments, and recommendations about the manuscript:

1. In-text referencing: Some sentences needs to be referenced e.g. Paragraph 2 (Page 4) has some sentences with facts that needs to be referenced accordingly.
2. Paragraph 1 (Page 5) presents a review on a study by Msoka et al. (2017). Please, change the first word in sentence 2 "A"  to "The"
3. On the "International Evidence of Processing of cashew apple", it must be noted that some African countries are doing well regarding its usage/applications; e.g., Nigeria. The authors must provide a paragraph on some of these countries and their contributions.
4. Please add separate section dealing with various factor and challenges (technical) that can be faced during industrial scale processing/application of cashew apples/fruits.
5. I suggest authors to write future perspectives on the cashew apple processing
6. I should commend the authors on the "actionable recommendations". However, they failed to specify who/which organization or party is responsible for the implementation

Does the paper provide a comprehensive overview of the policy and the context of its implementation in a way which is accessible to a general reader?

Yes

Is the discussion on the implications clearly and accurately presented and does it cite the current literature?

Partly

Are the recommendations made clear, balanced, and justified on the basis of the presented arguments?

Partly

Reviewer Expertise:

Waste and Biomass valorisation; Industrial Chemistry, Environmental Chemistry

I confirm that I have read this submission and believe that I have an appropriate level of expertise to confirm that it is of an acceptable scientific standard, however I have significant reservations, as outlined above.

# Response

**Published:** 2024-03-24

**Authors:** Noel Dimoso

**Affiliations:** Nelson Mandela African Institution of Science and Technology, Tanzania

1. A suggested in-text reference is included in this version

 2. A suggested correction was addressed in this version

 3. On the "International Evidence of Processing of cashew apple" section, more cashew apple startups from different African countries were added in this version

 4. A separate section dealing with various factor and challenges (technical) that can be faced during industrial scale processing/application of cashew apples/fruits is included in this version

 5. A new section 'future perspectives on the cashew apple processing' is included in this version

 6. A section 'Actionable recommendation' is re-written to address an organization or party that will implement those recommendations

# Reviewer report: Reviewer response for version 1

**DOI:** 10.5256/f1000research.136803.r203422

**Published:** 2023-09-26

**Authors:** Iheanyi William Eke

**Affiliations:** 1 Department of Pure & Industrial Chemistry, University of Port Harcourt, Choba, Rivers, Nigeria

The paper reviews the current gaps in the cashew apple value chain in Tanzania and proposes some policy-based solutions to change the status quo for sustainable development.

 Minor revision is recommended before publication:

1. Some important references are missing. For example (i) Page 5 paragraph 2 "A study revealed a good quantity of...." (which study?)
  (ii) Page 4 "A large segment (75 percent) of Tanzania’s population is under the age of 35 years. This young generation faces a great challenge to land employment immediately after finishing their qualifications. Statistically, out of one million young people, only 20 percent get employed immediately" (reference?) etc.
2. In the section "Actionable Recommendations", it will be useful to include the relevant action party (ies) in Tanzania to see to the implementation of these recommendations. Furthermore, the authors should proffer some practical steps/activities which these action parties could take/undertake towards the implementation of these recommendations. For instance, who is the action party in "encouraging and supporting youth and women to venture into the cashew apple value chain", and what are some of the steps this action party could take to achieve this. This same guideline should be applied to other recommendations. This will make them more actionable.

Does the paper provide a comprehensive overview of the policy and the context of its implementation in a way which is accessible to a general reader?

Partly

Is the discussion on the implications clearly and accurately presented and does it cite the current literature?

Yes

Are the recommendations made clear, balanced, and justified on the basis of the presented arguments?

Partly

Reviewer Expertise:

Biomass valorization

I confirm that I have read this submission and believe that I have an appropriate level of expertise to confirm that it is of an acceptable scientific standard, however I have significant reservations, as outlined above.

# Response

**Published:** 2024-03-24

**Authors:** Noel Dimoso

**Affiliations:** Nelson Mandela African Institution of Science and Technology, Tanzania

1. All important references were addressed in the new version as suggested

 2. The section "Actionable Recommendations", is re-written in the new version to include the relevant action party (ies) in Tanzania to see to the implementation of these recommendations.
